# Paravertebral Versus EPidural Analgesia in Minimally Invasive Esophageal ResectioN (PEPMEN): A Randomized Controlled Multicenter Trial

**DOI:** 10.1097/SLA.0000000000006551

**Published:** 2024-10-03

**Authors:** Minke L. Feenstra, Cezanne D. Kooij, Wietse J. Eshuis, Eline M. de Groot, Jeroen Hermanides, B. Feike Kingma, Suzanne S. Gisbertz, Jelle P. Ruurda, Freek Daams, Marije Marsman, Oscar F.C. van den Bosch, Werner ten Hoope, Lucas Goense, Misha D.P. Luyer, Grard A.P. Nieuwenhuijzen, Harm J. Scholten, Marc Buise, Marc J. van Det, Ewout A. Kouwenhoven, Franciscus van der Meer, Geert W.J. Frederix, Markus W. Hollmann, Edward Cheong, Mark I. van Berge Henegouwen, Richard van Hillegersberg

**Affiliations:** * Department of Surgery, Amsterdam UMC Location University of Amsterdam, AGEM, Cancer Center Amsterdam, Amsterdam, The Netherlands; † Department of Anesthesiology, Amsterdam UMC location University of Amsterdam, Amsterdam, The Netherlands; ‡ Department of Surgery, University Medical Center Utrecht, Utrecht, The Netherlands; § Department of Anesthesiology, University Medical Center Utrecht, Utrecht, The Netherlands; ∥ Department of Anesthesiology, Amsterdam UMC location Vrije Universiteit Amsterdam, Amsterdam, The Netherlands; ¶ Department of Surgery, Catharina Hospital, Eindhoven, The Netherlands; # Department of Anesthesiology, Catharina Hospital, Eindhoven, The Netherlands; ** Department of Anesthesia and Pain Medicine, Maastricht University Medical Center, Maastricht, The Netherlands; †† Department of Surgery, Hospital Group Twente, Almelo, The Netherlands; ‡‡ Department of Anesthesiology, Hospital Group Twente, Almelo, The Netherlands; §§ Department of Public Health, Healthcare Innovation & Evaluation and Medical Humanities, University Medical Center Utrecht, Utrecht, The Netherlands; ∥∥ Department of Upper GI, PanAsia Surgery, Singapore

**Keywords:** epidural analgesia, esophageal cancer, minimally invasive esophagectomy, paravertebral analgesia

## Abstract

**Objective:**

To compare the quality of recovery in patients receiving epidural or paravertebral analgesia for minimally invasive esophagectomy (MIE).

**Background:**

Paravertebral analgesia may be a promising alternative to epidural analgesia, avoiding potential side effects and improving postoperative recovery.

**Methods:**

This randomized controlled superiority trial was conducted across 4 Dutch centers with esophageal cancer patients scheduled for transthoracic MIE with intrathoracic anastomosis, randomizing patients to receive either epidural or paravertebral analgesia. The primary outcome was Quality of Recovery (QoR-40) on the third postoperative day (POD). Secondary outcomes included quality of life, postoperative pain, opioid consumption, inotropic/vasopressor medication use, hospital stay, complications, readmission, and mortality.

**Results:**

From December 2019 to February 2023, 192 patients were included: 94 received epidural and 98 paravertebral analgesia. QoR-40 score on POD3 was not different between groups (mean difference: 3.7, 95% CI: −2.3 to 9.7; *P*=0.268). Epidural patients had significantly higher QoR-40 scores on POD1 and 2 (mean difference: 7.7, 95% CI: 2.3–13.1; *P*=0.018 and mean difference: 7.3, 95% CI: 1.9–12.7; *P*=0.020) and lower pain scores (median 1 vs 2; *P*<0.001 and median 1 vs 2; *P*=0.033). More epidural patients required vasopressor medication on POD1 (38.3% vs 13.3%; *P*<0.001). Urinary catheters were removed earlier in the paravertebral group (median POD3 vs 4; *P*<0.001). No significant differences were found in postoperative complications or hospital/intensive care unit stay.

**Conclusions:**

This randomized controlled trial did not demonstrate the superiority of paravertebral over epidural analgesia regarding the quality of recovery on POD3 after MIE. Both techniques are effective and can be offered in clinical practice.

Esophageal cancer is the eighth most commonly diagnosed cancer and the sixth most common cause of death from cancer worldwide.^1–4^ Advances in (neo)adjuvant and surgical treatment strategies have substantially improved survival rates for locally advanced esophageal cancer, making multimodal treatment the standard of care in most centers worldwide.^5,6^ The traditional open esophagectomy is associated with significant postoperative pain, predominantly due to the thoracotomy.^7–9^ Inadequate pain management can lead to immobility, pulmonary complications, and prolonged hospitalization.^10,11^ Minimally invasive esophagectomy (MIE) has demonstrated lower postoperative complication rates, less pain, and faster recovery.^8,12,13^ With reduced postoperative pain in minimally invasive surgery, pain management may also be adjusted accordingly.^14^

Thoracic epidural analgesia has been the mainstay for pain control in patients undergoing open esophagectomy.^15,16^ However, its superiority over other pain management strategies with regard to pain control after esophagectomy remains inconclusive in meta-analyses.^10,17^ Furthermore, the rise of MIE occurred simultaneously with the progressive implementation of enhanced recovery after surgery (ERAS) protocols in the perioperative care of patients undergoing esophagectomy, which have also been shown to accelerate recovery.^18,19^ Although effective epidural analgesia potentially contributes to achieving ERAS goals in patients undergoing MIE, it can have counterproductive side effects due to motor and autonomic blockade (37% to 80%), resulting in hypotension, urinary retention, and reduced mobility.^20,21^ Some studies suggest that epidural analgesia might contribute to anastomotic leakage due to intraoperative hypotensive episodes.^22,23^ Moreover, epidural analgesia failure occurs in up to 30% of patients, often due to incorrect placement and migration after initial correct placement.^24^ In MIE, epidural analgesia has even been described as insufficient in up to half of patients.^25^ Furthermore, epidural analgesia may be associated with severe complications including epidural hematoma (1:4362) or abscess (1:10000), which can lead to permanent neurological damage, accidental high block and dural puncture.^26–29^

Paravertebral analgesia emerges as a promising alternative, utilizing unilateral catheter placement in the paravertebral space to infuse local anesthetics, demonstrating fewer side effects and complications than epidural analgesia.^30,31^ Though systematic reviews comparing paravertebral with epidural analgesia for patients undergoing open thoracic surgery show similar pain relief with fewer side effects for paravertebral analgesia, the evidence is lacking for patients undergoing MIE.^32,33^ Therefore, this study compares the postoperative quality of recovery in patients receiving epidural or paravertebral analgesia for MIE. We hypothesize that paravertebral analgesia provides a better quality of recovery than epidural analgesia, due to similar pain control and a lower incidence of side effects that may hinder postoperative recovery.

## METHODS

### Trial Design

This was an open randomized controlled superiority trial comparing epidural with paravertebral analgesia in 4 Dutch centers: (1) University Medical Center Utrecht, (2) Amsterdam UMC, (3) Catharina Hospital Eindhoven, and (4) Hospital Group Twente Almelo. The study protocol was approved by the institutional review board at each center and published previously (Dutch Trial Register: NL8037).^34^ The reporting of this study adhered to the CONSORT statement.^35^

### Participants

Adult patients who were scheduled to undergo 2-stage elective conventional or robot-assisted MIE with 2-field lymphadenectomy, gastric conduit reconstruction, and an intrathoracic anastomosis (Ivor Lewis procedure) were included. The procedure consists of a laparoscopic abdominal phase with mobilization of the stomach, abdominal lymphadenectomy, and construction of a gastric conduit; and then, a right-sided thoracoscopic phase, during which mobilization of the esophagus, mediastinal lymphadenectomy, and an intrathoracic anastomosis is performed.

Exclusion criteria were severe comorbidity (ASA>III), contraindications for epidural analgesia,^36^ allergy to local anesthetics, ongoing opioid use (>3 months prior to the day of surgery), renal failure (eGFR <50 mL/min), inability to provide informed consent or complete questionnaires in Dutch and cervical lymph node dissection.

### Randomization and Masking

Written informed consent was obtained, after initial screening for eligibility at the preoperative outpatient clinic. After inclusion, and ultimately on the day prior to surgery, patients were entered into the data capturing platform Castor Electronic Data Capture, which includes a digital randomization tool used to assign patients to either the epidural or paravertebral analgesia group.^37^ Randomization was stratified per center. The random assignment was performed in a 1:1 ratio using block randomization (block sizes 2, 4, and 6). Patients and physicians were not blinded for group allocation.

### Procedures

#### Epidural Regimen

Before anesthesia induction, an epidural catheter was placed at an intervertebral, mid-thoracic level (T5–T8), with the needle directed cranially aiming to position the catheter at level T4–T5. Within the first hour after induction, a bolus of 5 to 10 mL of bupivacaine 0.25% was administered. Continuous epidural analgesia was started (bupivacaine 0.125% + sufentanil 0.5 mcg/mL) and postoperatively continued with an infusion of 6 to 14 mL/h and titrated to the patient’s comfort. Escape medication was provided according to the local protocol of the participating center. The aim was to remove the epidural catheter on the third postoperative day (POD).

#### Paravertebral Regimen

Intraoperatively, at the start of the thoracic phase, a paravertebral catheter was placed by the surgeon in the right subpleural space at the level of T4–T5 under direct thoracoscopic vision (Video 1). After an initial bolus of 20 mL bupivacaine 0.125%, a continuous infusion of 8 to 12 mL/h was started after the procedure, depending on weight, and titrated to the patient’s comfort. Patient-controlled intravenous analgesia was additionally provided according to the local protocol of the participating center. The aim was to remove the paravertebral catheter on POD3.

**Video 1:** Paravertebral catheter placement procedure.

#### Quality Control

Prior to the start of patient inclusion, participating surgeons without previous experience with paravertebral catheterizations performed their first paravertebral catheterization under the guidance of a surgeon with vast experience with this analgesia technique (E.C., M.D.P.L, and M.I.v.B.H.). The surgeons had to perform at least 3 successful paravertebral catheterizations before starting inclusion. During the trial, paravertebral procedures were video recorded and stored for quality control by an expert (E.C.) for feedback. Procedures were scored (scale 1–3).

### Outcomes

The primary outcome was the total score of the Quality of Recovery (QoR-40) questionnaire on the morning of POD3. The QoR-40 is a validated and suitable patient-reported outcome measure (PROM) of postoperative quality of recovery (Supplemental Methods, Supplemental Digital Content 1, http://links.lww.com/SLA/F324). Secondary outcomes were QoR-40 score on POD1–2, pain scores on POD1–3 according to the numeric rating scale (NRS), postoperative pain experience according to the International Pain Outcomes (IPO)-questionnaire on POD1–3 (Supplemental Methods, Supplemental Digital Content 1, http://links.lww.com/SLA/F324), duration of anesthesia, duration of surgery time, the use of escape pain medication on POD1–3, total opioid consumption [administered via epidural analgesia catheter, intravenously or orally; in oral morphine equivalents (OME)] on POD1–3, technical complications, effort of catheter placement according to the Subjective Mental Effort Questionnaire (SMEQ) (Supplemental Methods, Supplemental Digital Content 1, http://links.lww.com/SLA/F324), analgesia-related side effects, need for inotropic or vasopressor medication, fluid balance, length of stay on an intensive unit (ICU) or medium care unit (MCU) and in hospital stay, POD of removal of the urinary catheter, POD of removal of the epidural or paravertebral catheter and mobilization. Postoperative complications (according to the Esophageal Complications Consensus Group and their corresponding Clavien-Dindo classification; Supplemental Methods, Supplemental Digital Content 1, http://links.lww.com/SLA/F324) and readmission were documented until POD30, mortality was documented until POD90.

### Statistical Analysis

#### Sample Size

To detect a minimally clinically important difference (MCID) in the QoR-40 of 6.3 points^38^ with an estimated SD of 14, α of 0.05 and power of 0.8, 172 patients were needed for the independent *T* test.^39,40^ Anticipating a 10% loss to follow-up, 192 patients had to be included. Dropouts before surgery and screening failures were replaced.

#### Statistics

All analyses were performed using IBM SPSS Statistics (Version 28). An intention-to-treat analysis was performed to analyze the between group difference in primary outcome using an independent *T* test. In addition, a per-protocol analysis was performed. A post hoc analysis was performed to account for missing items in the QoR-40 questionnaire.^41^ Missing QoR-40 values were encountered in 36, 26, 28, and 29 of patients on the preoperative day and POD1–3, respectively. Missing data were considered at random and handled using multiple imputations with the iterative Markov chain Monte Carlo method creating 20 data sets. To handle the high number of questionnaire variables in the imputation model, parcel summary scores of the questionnaire data were created.^41^ A rationale and description of this method is shown in Supplemental Methods, Supplemental Digital Content 1, http://links.lww.com/SLA/F324. Analyses of secondary outcomes were performed with χ^2^ tests for categorical data. Continuous outcomes with a normal or non-normal distribution were compared using the *T* test or Mann-Whitney *U* test, respectively. To correct for multiple testing, the Benjamini-Hochberg method was applied.^42^

## RESULTS

**FIGURE 1:**
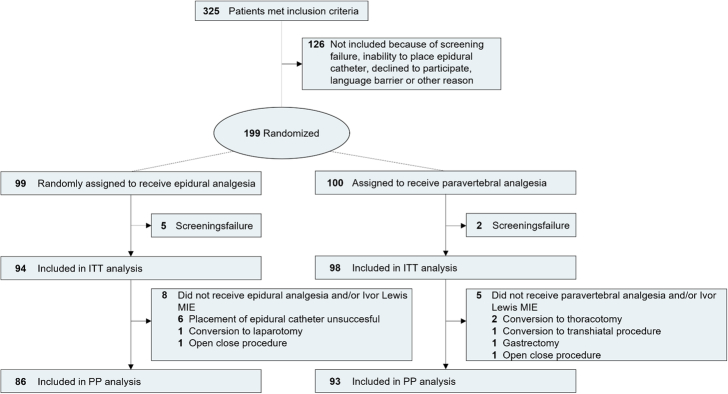
Trial flowchart. Of the 199 patients who underwent random assignment, 192 patients were included in the intention-to-treat analysis: 94 in the epidural group and 98 in the paravertebral group. A total of 179 patients underwent Ivor Lewis minimally invasive esophagectomy and their allocated analgesia modality according to the protocol: 86 in the epidural group and 93 in the paravertebral group. MIE indicates minimally invasive esophagectomy; ITT, intention-to-treat; PP, per protocol.

From December 2019 to June 2022, 325 patients met the inclusion criteria and eventually 192 patients were included in the intention-to-treat analysis: 94 patients were assigned to the epidural, and 98 patients to the paravertebral group (Fig. 1). The included patients had a median age of 67 years and 82% were male (Table 1). Of these patients, 86 (91.5%) from the epidural group and 93 (94.9%) from the paravertebral group were treated according to the protocol. The reasons for deviations are shown in Figure 1. Intraoperative details are shown in the Supplemental Results (Supplemental Digital Content 1, http://links.lww.com/SLA/F324).

**TABLE 1:** Patient Characteristics at Baseline

	Epidural (n=94)	Paravertebral (n=98)
Age, mean (SD), y	65.2 (8.9)	66.3 (8.8)
No. (%) male sex	73 (77.7)	84 (85.7)
BMI, mean (SD), kg/m^2^	26.7 (±4.5)	25.7 (±3.4)
ASA score, No. (%)		
1	4 (4.3)	1 (1.0)
2	61 (64.9)	65 (66.3)
3	29 (30.9)	32 (32.7)
No. (%) with cardiovascular comorbidity	19 (20.2)	20 (20.4)
No. (%) with pulmonary comorbidity	13 (13.8)	14 (14.3)
No. (%) with diabetes mellitus	12 (12.8)	14 (14.3)
No. (%) with hypertension	33 (35.1)	38 (38.8)
No. (%) with opioid use <3 months	2 (2.1)	3 (3.1)

ASA indicates American Society of Anesthesiologists; BMI, body mass index.

### Primary Outcome

The QoR-40 questionnaire on POD3 was completed in the epidural group by 91 patients (97%) and in the paravertebral group by 98 patients (100%) patients. Of the 189 patients who completed the questionnaire, 26 had ≥1 missing item(s). In both the intention-to-treat analysis and the per-protocol analysis, the quality of recovery on POD3 did not differ between the epidural versus the paravertebral group (168.8 vs 165.1, mean difference: 3.7, 95% CI: −2.3 to 9.7; *P*=0.268 and 168.8 vs 165.2, mean difference: 3.6, 95% CI: −2.6 to 9.9; *P*=0.273, respectively). Primary outcome did not change after imputing missing data (168.6 vs 163.2, 95% CI: −0.0 to 10.8; *P*=0.093) (Table 2).

**TABLE 2:** Quality of Recovery (QoR)-40 Measured Preoperatively and Postoperatively

	Epidural		Paravertebral					
	N	Mean QoR-40	N	Mean QoR-40	Mean difference^*^	95% CI	*P*	*P* ^†^
POD3								
ITT CC	83	168.8	80	165.1	3.7	−2.3, 9.7	0.227	0.268
ITT imputed^‡^	94	168.6	98	163.2	5.4	−0.0, 10.8	0.050	0.093
PP CC	76	168.8	77	165.2	3.6	−2.6, 9.9	0.252	0.273
Pre-op								
ITT CC^§^	79	177.2	77	174.2	3.4	−0.3, 7.1	0.069	0.100
ITT imputed^‡^ ^§^	94	175.2	98	172.2	2.0	−2.0, 5.9	0.302	0.302
PP CC	72	179.6	73	176.4	3.2	−0.7, 7.2	0.116	0.151
POD1								
ITT CC	84	168.8	82	161.3	7.7	2.3, 13.1	**0.005**	**0.018**
ITT imputed^‡^	94	168.3	98	161.2	7.1	2.0, 12.3	**0.007**	**0.018**
PP CC	78	169.2	79	161.1	7.8	2.2, 13.4	**0.007**	**0.018**
POD2								
ITT CC	84	168.8	78	161.5	7.3	1.9, 12.7	**0.009**	**0.020**
ITT imputed^‡^	94	169.0	98	160.0	8.9	4.2, 13.7	**<0.001**	**<0.001**
PP CC	78	168.9	74	161.0	8.0	2.3, 13.6	**0.006**	**0.018**
AUC								
ITT CC	74	339.7	61	329.3	10.4	−5.0, 21.3	0.061	0.099


Bold values are statistically significant.

^*^ Minimally clinically important difference (MCID): 6.3 (Myles et al, 2016^38^).

^†^ *P* values were corrected with the Benjamini-Hochberg method.

^‡^ Multiple imputation with parcel summary scores.

^§^ Three questions excluded (>50% missing values, QoR 16, 17, and 18).

AUC indicates area under the curve analysis; CC, complete case analysis; ITT, intention-to-treat analysis; POD, postoperative day; PP, per protocol analysis; preop, preoperative.

### Quality Control

Before the trial, all participating surgeons of the 4 centers followed the training in paravertebral catheter placement and performed 3 successful procedures, each under expert guidance (E.C.). During the trial, an expert (E.C.) assessed and scored 77.6% (76 of 98) of paravertebral catheter placements, providing feedback for any deviations. The remaining paravertebral procedures could not be assessed due to the absence of video recordings. The mean overall quality score (scale 1–3) was 3, indicating a “good” rating. No inter-hospital differences were noted.

### Secondary Outcomes

#### QoR-40 and Pain

Table 2 displays QoR-40 results for POD1–3. On POD1 and 2, the epidural group showed significantly higher QoR-40 scores (mean difference of 7.7 on POD1, 95% CI: 2.3–13.1; *P*=0.018 and 7.3 on POD2, 95% CI: 2.3–13.6; *P*=0.018, respectively) and lower NRS pain scores [median 1 (IQR: 0–3) vs 2 (IQR: 1–3); *P*<0.001 on POD1 and median 1 (IQR: 0–2) vs 2 (IQR: 0–3); *P*=0.033 on POD2] (Supplemental Table 1, Supplemental Digital Content 1, http://links.lww.com/SLA/F324). On POD3, NRS pain scores were not significantly different [median NRS 1 (IQR: 0–3) vs NRS 2 (IQR: 0–3); *P*=0.315]. The IPO questionnaires revealed similar results, as more pain was reported by the paravertebral group on POD1 and 2. Both groups reported similar satisfaction with their pain management on POD3 (Supplementary Table 2, Supplemental Digital Content 1, http://links.lww.com/SLA/F324). The pain was more often located in the abdomen in the paravertebral group compared with the epidural group on POD1 (22.4% vs 8.5%; *P*=0.018) and POD2 (20.4% vs 9.6%; *P*=0.050). On POD3, no difference in pain localization was observed between groups, but pain more often interfered with physical activities (taking deep breaths/coughing, sitting up) in the paravertebral group.

In the epidural group, opioids were administered via the epidural analgesia catheter, intravenously, and/or orally, while in the paravertebral group opioids were only administered intravenously and/or orally. Intraoperatively, there were no significant differences in total opioid consumption between the epidural and the paravertebral groups [median OME 1371 (IQR: 388–2442) vs 1790 (IQR: 345–2610); *P*=0.774] (Supplemental Table 3, Supplemental Digital Content 1, http://links.lww.com/SLA/F324). The total opioid consumption on POD1–3 was higher in the epidural group (*P*<0.001), as demonstrated in Supplemental Table 4, Supplemental Digital Content 1, http://links.lww.com/SLA/F324. There was no difference in postoperative mobilization (Supplemental Table 5, Supplemental Digital Content 1, http://links.lww.com/SLA/F324). However, as patients became more mobile, documenting activity levels decreased, leading to more missing data.

#### Epidural and Paravertebral Catheter Placement and Removal

Of the 94 attempted epidural catheter placements, 6 were unsuccessful due to the inability to navigate between the vertebrae, patient movement, or vagal responses. Of these 6 patients, 5 were subsequently given a paravertebral catheter intraoperatively, while 1 did not receive a catheter and only received analgesia intravenously. Of the patients assigned to receive a paravertebral catheter, 3 did not have the catheter placed due to conversion to a transhiatal procedure, the performance of a gastrectomy, and an open-close procedure. In one of them, an epidural catheter was placed on POD1. No difference was reported in terms of effort of analgesia catheter placement [median SMEQ-score 40 (IQR: 12–75) for epidural vs 30 (IQR: 20–49) for paravertebral placement; *P*=0.397] (Supplemental Table 3, Supplemental Digital Content 1, http://links.lww.com/SLA/F324). The median duration of epidural catheter placement was longer than paravertebral placement [15 minutes (IQR: 12–19) vs 5 minutes (IQR: 4–8); *P*<0.001].

Premature removal of the catheter (before POD3) in the patients who were treated according to protocol occurred in 12 of 86 epidural catheters (14.0%) and 15 of 93 paravertebral catheters (16.1%), mostly due to dislocation and ineffective pain management. In 3 patients, all within the epidural group, the catheter had to be removed because of hypotension. No infectious or bleeding complications associated with the placement of epidural or paravertebral catheters, such as hematomas or abscesses, were observed. In both groups, the median POD of analgesia catheter removal was POD3. The urinary catheter was removed one day earlier in paravertebral analgesia [median POD3 (IQR: 2–3) vs POD4 (IQR: 3–4); *P*<0.001].

#### Fluid Balance and Vasopressor Medication

No differences were reported between the epidural and paravertebral groups in duration of anesthesia, vasopressor use, and fluid balance intraoperatively (Supplementary Table 3, Supplemental Digital Content 1, http://links.lww.com/SLA/F324). On POD1–3, no difference in fluid administration between the 2 groups on POD1–3 was noted (Supplemental Table 6, Supplemental Digital Content 1, http://links.lww.com/SLA/F324). In the epidural group, more patients required vasopressors on POD1 (38.3% vs 13.3%; *P*<0.001). Of those patients who required vasopressors (norepinephrine) in both the epidural and paravertebral group, there was no difference in the total administered dose between the 2 groups.

#### Postoperative Outcomes

As shown in Table 3, postoperative complications did not differ between the epidural and paravertebral groups, specifically regarding anastomotic leakage (13 patients; 13.8% vs 6 patients; 6.1%; *P*=0.828) and pneumonia (11.7% vs 18.4%; *P*=0.853). Length of stay on a monitored unit was similar in the groups [median 1 day (IQR: 1–1) vs 1 day (IQR: 1–1); *P*=0.853]. Length of hospital stay was also comparable [median 8 days in the epidural group (IQR: 7–11) vs 8 days in the paravertebral group (IQR: 7–13); *P*=0.853]. In the epidural group, 2 patients (2.1%) died in hospital due to anastomotic leakage and sepsis. In the paravertebral group, no patients died in the hospital.

**TABLE 3:** Postoperative Complications and Outcomes

	Epidural (n=94)	Paravertebral (n=98)	*P* ^*^	*P* ^†^
No. (%) with overall complications	53 (56.4)	53 (54.1)	0.749	0.853
No. (%) with anastomotic leakage	13 (13.8)	6 (6.1)	0.092	0.828
No. (%) with pulmonary complication	27 (28.7)	26 (26.5)	0.749	0.853
No. (%) with pneumonia	11 (11.7)	18 (18.4)	0.230	0.853
No. (%) with cardiac complication	15 (16.0)	18 (18.4)	0.705	0.853
No. (%) with urinary tract infection	1 (1.1)	1 (1.0)	1.000	1.000
No. (%) of deaths	5 (5.3)	4 (4.1)		
No. (%) of in-hospital deaths	2 (2.1)	0 (0.0)		
No. (%) of deaths within 90 POD	3 (3.2)	4 (4.1)	0.333	0.853
ICU/MCU stay, median (IQR), d	1 (1–1)	1 (1–1)	0.758	0.853
Hospital stay, median (IQR), d	8 (7–11)	8 (7–13)	0.575	0.853

^*^ Calculated with the χ^2^ test, in case of the median (IQR) calculated with Mann-Whitney *U* test.

^†^ *P* values were corrected with the Benjamini-Hochberg method.

ICU indicates intensive care unit; IQR, interquartile range; MCU, medium care unit; POD, postoperative day.

## DISCUSSION

This multicenter randomized controlled trial compared epidural with paravertebral analgesia in patients undergoing MIE. The quality of recovery on POD3 was similar in both groups. In the secondary analyses, the paravertebral group was found to report lower quality of recovery on POD1 and 2, as well as to experience more interference with activities due to pain. On the other hand, patients with epidural analgesia required more vasopressors on POD1 and had an extra day of urinary catheterization. Despite these differences, there were no significant variations in postoperative complications or length of high care/hospital stay. Based on these findings, either analgesia regimen is appropriate for clinical use, with the decision depending on the preferences of the physician and patient.

This is the first randomized clinical trial comparing epidural with paravertebral analgesia in MIE. Our primary outcome (QoR-40 on POD3), was similar in both groups (no significant nor clinically relevant difference). The QoR-40, a widely recognized PROM, has gained a prominent role in clinical trials as it provides a multifaceted view of recovery. Personal communication before the trial with the developer of the questionnaire suggested that POD3 was the optimal time to differentiate any beneficial effects. The total QoR-40 score exhibits a strong correlation with both postoperative pain and length of hospital stay.^40^ Patients who received epidural analgesia had slightly higher QoR-40 scores on POD1 and 2, with a difference just exceeding the MCID. The statistically significant differences in NRS pain scores on POD1 and 2 are not considered clinically relevant, which is in line with a recent Cochrane review comparing epidural with paravertebral analgesia in thoracic surgery.^32^ Notably, the Cochrane review did not include patient-reported pain outcomes. In our trial, better PROMs were identified with epidural analgesia using the IPO-questionnaire, consistent with a retrospective study conducted by our group showing lower pain scores in patients esophagectomy with intrathoracic anastomosis and epidural analgesia.^43^ Opioid consumption was higher in the epidural group, which was inherent to the study protocol, as the epidural group received continuous epidural opioid infusion while patients in the paravertebral group had patient-controlled intravenous opioid administration with a bolus function. The standard of care is epidural analgesia with local anesthetic and an opioid. In this trial, we compared this standard of care with paravertebral analgesia. Since there are no known receptors for opioids in the paravertebral space, administering continuous opioids through the paravertebral catheter would be ineffective. However, this setup complicates the direct comparison of opioid consumption between the groups. Future research could explore a comparison between local-only epidural and local-only paravertebral blocks to address this issue.

The urinary catheter was removed one day earlier in the paravertebral group, aligning with the ERAS protocol. Possibly, earlier removal contributes to patient comfort and, consequently, quality of recovery. However, since the urinary catheter was removed on POD3 in the paravertebral and on POD4 in the epidural group, this aspect was most likely not measured by the QoR-40 on POD3.

Importantly, all participating centers of this multicenter trial adhered to an ERAS protocol aimed at optimizing patient recovery. Although the guidelines within these protocols are largely uniform, some variation between centers may exist regarding drain placement and the intensity of mobilization and/or physiotherapy. It is worth noting that such variations are independent of the randomization group assignment.

Consistent with the Cochrane review, our trial found no significant differences in mortality, major complications, or length of hospital/ICU stay between the 2 analgesia groups.^32^ The length of hospital stay likely hinges on factors beyond analgesia modality, such as postoperative complications or physical fitness. Notably, overall complication rates were similar between groups. While leakage of the esophagogastric anastomosis was higher in the epidural group (13.8% vs 6.1%), this difference did not reach statistical significance. It is important to note that our study was not powered to detect differences in anastomotic leakage rates, but this finding suggests a need for further investigation into the potential association between epidural analgesia and anastomotic leakage. Current literature on this topic is not conclusive.^23,44^ While one retrospective study reported increased anastomotic leakage with intraoperative epidural analgesia, other retrospective propensity score-matched studies have not supported these findings.^20,22,45,46^

In this trial, several quality control measures were implemented in the design. The results demonstrate that the placement of the paravertebral catheter was considered straightforward and safe. Moreover, the majority of paravertebral catheter insertions were considered to exhibit good overall quality.

This study has some limitations. First, missing data in the questionnaire for our primary outcome occurred. Though common, we did not specify an imputation plan in the protocol. We used a validated imputation method, mitigating potential bias. Second, we opted for a continuous epidural infusion without a patient-controlled bolus function. However, patients in the paravertebral group who did have a patient-controlled bolus function (albeit intravenously), might therefore have experienced greater autonomy, potentially influencing their quality of recovery. Not all participating centers used patient-controlled functions on the epidural before the trial, which is why it is not standard of care in the trial. Importantly, there is no evidence supporting the superiority of patient-controlled bolus with continuous infusion over continuous infusion alone in thoracic surgery. Furthermore, it is important to acknowledge that the design of this study was a superiority trial, whereas a noninferiority trial might have been more appropriate.

A notable strength of this study is the quality control implemented for paravertebral catheter insertions. Moreover, as a randomized controlled trial conducted in 4 high-volume centers with a participant pool representative of esophageal cancer patients in the Western world, our findings ensure generalizability to the experiences in Western high-volume centers.

In conclusion, this multicenter randomized controlled clinical trial did not show the superiority of paravertebral analgesia over epidural analgesia in the quality of recovery on the third day after minimally invasive esophagectomy. The paravertebral group had a lower quality of recovery on POD1 and 2. In the epidural group, more patients consumed vasopressor medication on POD1 and had a prolonged urinary catheterization by one day. However, these factors did not result in extended stays in a high-care unit or in the hospital. There were no statistically significant differences in postoperative complications. These results, however, support the safety of paravertebral analgesia as a viable alternative to epidural analgesia, enabling the provision of both techniques to patients in clinical practice.

## Supplementary Material

## References

[1] DiSiena M Perelman A Birk J . Esophageal cancer: an updated review. South Med J. 2021;114:161–168. 33655310 10.14423/SMJ.0000000000001226

[2] Yibulayin W Abulizi S Lv H . Minimally invasive oesophagectomy versus open esophagectomy for resectable esophageal cancer: a meta-analysis. World J Surg Oncol. 2016;14:304. 27927246 10.1186/s12957-016-1062-7 PMC5143462

[3] Enzinger PC Mayer RJ . Esophageal cancer. N Engl J Med. 2003;349:2241–2252. 14657432 10.1056/NEJMra035010

[4] Morgan E Soerjomataram I Rumgay H . The global landscape of esophageal squamous cell carcinoma and esophageal adenocarcinoma incidence and mortality in 2020 and projections to 2040: new estimates from GLOBOCAN 2020. Gastroenterology. 2022;163:649–658.e2. 35671803 10.1053/j.gastro.2022.05.054

[5] Lagergren J Smyth E Cunningham D . Oesophageal cancer. Lancet. 2017;390:2383–2396. 28648400 10.1016/S0140-6736(17)31462-9

[6] Elliott JA Markar SR Klevebro F . An international multicenter study exploring whether surveillance after esophageal cancer surgery impacts oncological and quality of life outcomes (ENSURE). Ann Surg. 2022;277:e1035–e1044. 35129466 10.1097/SLA.0000000000005378 PMC10082056

[7] Yu G Cheng X Jin C . Clinical effect and postoperative pain of laparo-thoracoscopic esophagectomy in patients with esophageal cancer. Evid Based Complement Alternat Med. 2022;2022:1–7. 10.1155/2022/4507696 PMC9251098 35795286

[8] Watson TJ . Open’ esophagectomy. J Gastrointest Surg. 2011;15:1500–1502. 21594700 10.1007/s11605-011-1560-4

[9] Furrer M Rechsteiner R Eigenmann V . Thoracotomy and thoracoscopy: postoperative pulmonary function, pain and chest wall complaints. Eur J Cardiothorac Surg. 1997;12:82–87. 9262085 10.1016/s1010-7940(97)00105-x

[10] Visser E Marsman M van Rossum P . Postoperative pain management after esophagectomy: a systematic review and meta-analysis. Dis Esophagus. 2017;30:1–11. 10.1093/dote/dox052 28859388

[11] Richardson J Sabanathan S Mearns AJ . Efficacy of pre-emptive analgesia and continuous extrapleural intercostal nerve block on post-thoracotomy pain and pulmonary mechanics. J Cardiovasc Surg (Torino). 1994;35:219–228. 8040170

[12] Biere SS van Berge Henegouwen MI Maas KW . Minimally invasive versus open oesophagectomy for patients with oesophageal cancer: a multicentre, open-label, randomised controlled trial. Lancet. 2012;379:1887–1892. 22552194 10.1016/S0140-6736(12)60516-9

[13] Yamamoto M Weber JM Karl RC . Minimally invasive surgery for esophageal cancer: review of the literature and institutional experience. Cancer Control. 2013;20:130–137. 23571703 10.1177/107327481302000206

[14] van der Sluis PC van der Horst S May AM . Robot-assisted minimally invasive thoracolaparoscopic esophagectomy versus open transthoracic esophagectomy for resectable esophageal cancer. Ann Surg. 2019;269:621–630. 30308612 10.1097/SLA.0000000000003031

[15] Durkin C Schisler T Lohser J . Current trends in anesthesia for esophagectomy. Curr Opin Anaesthesiol. 2017;30:30–35. 27764049 10.1097/ACO.0000000000000409

[16] Rudin Å Flisberg P Johansson J . Thoracic epidural analgesia or intravenous morphine analgesia after thoracoabdominal esophagectomy: a prospective follow-up of 201 patients. J Cardiothorac Vasc Anesth. 2005;19:350–357. 16130063 10.1053/j.jvca.2005.03.013

[17] Hughes M Yim I Deans DAC . Systematic review and meta-analysis of epidural analgesia versus different analgesic regimes following oesophagogastric resection. World J Surg. 2018;42:204–210. 28741191 10.1007/s00268-017-4141-1

[18] Low DE Allum W De Manzoni G . Guidelines for perioperative care in Esophagectomy: Enhanced Recovery After Surgery (ERAS®) Society Recommendations. World J Surg. 2019;43:299–330. 30276441 10.1007/s00268-018-4786-4

[19] Ashok A Niyogi D Ranganathan P . The enhanced recovery after surgery (ERAS) protocol to promote recovery following esophageal cancer resection. Surg Today. 2020;50:323–334. 32048046 10.1007/s00595-020-01956-1 PMC7098920

[20] Li W Li Y Huang Q . Short and long-term outcomes of epidural or intravenous analgesia after esophagectomy: a propensity-matched cohort study. PLoS One. 2016;11:e0154380. 27110939 10.1371/journal.pone.0154380 PMC4844138

[21] Liu S Carpenter RL Neal JM . Epidural anesthesia and analgesia. Anesthesiology. 1995;82:1474–1506. 7793661 10.1097/00000542-199506000-00019

[22] Fumagalli U Melis A Balazova J . Intra-operative hypotensive episodes may be associated with post-operative esophageal anastomotic leak. Updates Surg. 2016;68:185–190. 27146868 10.1007/s13304-016-0369-9

[23] Al-Rawi OY Pennefather SH Page RD . The effect of thoracic epidural bupivacaine and an intravenous adrenaline infusion on gastric tube blood flow during esophagectomy. Anesth Analg. 2008;106:884–887. 18292435 10.1213/ane.0b013e318164f153

[24] Hermanides J Hollmann MW Stevens MF . Failed epidural: causes and management. Br J Anaesth. 2012;109:144–154. 22735301 10.1093/bja/aes214

[25] Kingma BF Visser E Marsman M . Epidural analgesia after minimally invasive esophagectomy: efficacy and complication profile. Dis Esophagus. 2019;32. doi:10.1093/dote/doy116 30561659

[26] Bos EME Haumann J de Quelerij M . Haematoma and abscess after neuraxial anaesthesia: a review of 647 cases. Br J Anaesth. 2018;120:693–704. 29576110 10.1016/j.bja.2017.11.105

[27] Bos EME Hollmann MW Lirk P . Safety and efficacy of epidural analgesia. Curr Opin Anaesthesiol. 2017;30:736–742. 28938298 10.1097/ACO.0000000000000516

[28] Christie IW McCabe S . Major complications of epidural analgesia after surgery: results of a six-year survey. Anaesthesia. 2007;62:335–341. 17381568 10.1111/j.1365-2044.2007.04992.x

[29] Rosero EB Joshi GP . Nationwide incidence of serious complications of epidural analgesia in the United States. Acta Anaesthesiol Scand. 2016;60:810–820. 26876878 10.1111/aas.12702

[30] van den Berg JW Tabrett K Cheong E . Paravertebral catheter analgesia for minimally invasive Ivor Lewis oesophagectomy. J Thorac Dis. 2019;11(S5):S786–S793. 31080659 10.21037/jtd.2019.03.47 PMC6503262

[31] Coveney E Weltz CR Greengrass R . Use of paravertebral block anesthesia in the surgical management of breast cancer. Ann Surg. 1998;227:496–501. 9563536 10.1097/00000658-199804000-00008 PMC1191303

[32] Yeung JH Gates S Naidu BV . Paravertebral block versus thoracic epidural for patients undergoing thoracotomy. Cochrane Database Syst Rev. 2016;2016:CD009121. 10.1002/14651858.CD009121.pub2 PMC7151756 26897642

[33] Davies RG Myles PS Graham JM . A comparison of the analgesic efficacy and side-effects of paravertebral vs epidural blockade for thoracotomy—a systematic review and meta-analysis of randomized trials. Br J Anaesth. 2006;96:418–426. 16476698 10.1093/bja/ael020

[34] Kingma BF Eshuis WJ de Groot EM . Paravertebral catheter versus EPidural analgesia in Minimally invasive Esophageal resectioN: a randomized controlled multicenter trial (PEPMEN trial). BMC Cancer. 2020;20:142. 32087686 10.1186/s12885-020-6585-1 PMC7036230

[35] Schulz KF Altman DG Moher D . CONSORT 2010 Statement: updated guidelines for reporting parallel group randomised trials. Brit Med J. 2010;340:c332–c332. 20332509 10.1136/bmj.c332 PMC2844940

[36] Dutch Society for Anesthesiology, “Neuraxis and anti-coagulation,” Federatie Medisch Specialisten. Accessed October 16, 2023.https://richtlijnendatabase.nl/richtlijn/neuraxisblokkade_en_antistolling/neuraxisblokkade_en_antistolling_-_startpagina.html

[37] Castor Electronic Data Capture, “Castor EDC.” Accessed October 1, 2023. https://castoredc.com

[38] Myles PS Myles DB Galagher W . Minimal clinically important difference for three quality of recovery scales. Anesthesiology. 2016;125:39–45. 27159009 10.1097/ALN.0000000000001158

[39] Gornall BF Myles PS Smith CL . Measurement of quality of recovery using the QoR-40: a quantitative systematic review. Br J Anaesth. 2013;111:161–169. 23471753 10.1093/bja/aet014

[40] Myles PS Weitkamp B Jones K . Validity and reliability of a postoperative quality of recovery score: the QoR-40. Br J Anaesth. 2000;84:11–15. 10740540 10.1093/oxfordjournals.bja.a013366

[41] Eekhout I de Vet HC de Boer MR . Passive imputation and parcel summaries are both valid to handle missing items in studies with many multi-item scales. Stat Methods Med Res. 2018;27:1128–1140. 27334917 10.1177/0962280216654511

[42] Benjamini Y Hochberg Y . Controlling the false discovery rate: a practical and powerful approach to multiple testing. J R Stat Soc Series B Methodol. 1995;57:289–300.

[43] Feenstra ML ten Hoope W Hermanides J . Optimal perioperative pain management in esophageal surgery: an evaluation of paravertebral analgesia. Ann Surg Oncol. 2021;28:6321–6328. 34050429 10.1245/s10434-021-10172-1 PMC8460583

[44] Huisman DE Reudink M van Rooijen SJ . LekCheck: a prospective study to identify perioperative modifiable risk factors for anastomotic leakage in colorectal surgery. Ann Surg. 2022;275:e189–e197. 32511133 10.1097/SLA.0000000000003853 PMC8683256

[45] Hirano Y Kaneko H Konishi T . Short-term outcomes of epidural analgesia in minimally invasive esophagectomy for esophageal cancer: nationwide inpatient data study in Japan. Ann Surg Oncol. 2022;29:8225–8234. 35960454 10.1245/s10434-022-12346-x

[46] Wang W Zhao G Wu L . Risk factors for anastomotic leakage following esophagectomy: impact of thoracic epidural analgesia. J Surg Oncol. 2017;116:164–171. 28384375 10.1002/jso.24621

