# Supplementary material for: Paravertebral Versus EPidural Analgesia in Minimally Invasive Esophageal ResectioN (PEPMEN): A Randomized Controlled Multicenter Trial
Source: Ann Surg. 2024 Oct 3;282(1):29–36. doi: 10.1097/SLA.0000000000006551 (PMC12140555; doi:10.1097/SLA.0000000000006551)
Supplement: Supplementary file 2 [file sla-282-029-s001.docx]

**PEPMEN Supplementary Appendix**

**Table of Contents**

[**Supplementary methods** 2](#_Toc174111731)

[**Quality of Recovery 40** 2](#_Toc174111732)

[**Secondary outcome questionnaires and classifications** 2](#_Toc174111733)

[**Rationale and description of the imputation of the QoR-40 scores** 2](#_Toc174111734)

[**Supplementary results** 3](#_Toc174111735)

[**Supplementary table 1: Postoperative pain scores, expressed in numerical (pain) rating scale (NRS)** 3](#_Toc174111736)

[**Supplementary table 2: The IPO questionnaire POD 1, 2 and 3** 3](#_Toc174111737)

[**Supplementary table 3: Intraoperative outcomes** 5](#_Toc174111738)

[**Supplementary table 4: Postoperative opioid consumption** 6](#_Toc174111739)

[**Supplementary table 5: Mobilization** 6](#_Toc174111740)

[**Supplementary table 6: Fluid balance and vasopressor consumption intra- and postoperative** 7](#_Toc174111741)

[**References** 8](#_Toc174111742)

# **Supplementary methods**

## **Quality of Recovery 40**

The QoR-40 is a validated and suitable Patient Reported Outcome Measure (PROM) of postoperative quality of recovery. The QoR-40 contains 40 items, covering five dimensions: physical comfort, physical independence, psychological support, emotional state and pain. Each item can be rated 1 – 5, the total score ranges from 40 to 200, with a high score indicating a good quality of recovery.[1]

## **Secondary outcome questionnaires and classifications**

Secondary outcome questionnaires and classifications:

- International Pain Outcomes (IPO)-questionnaire [2]
- Effort of catheter placement according to the Subjective Mental Effort Questionnaire (SMEQ) [3]
- Postoperative complications according to the Esophageal Complications Consensus Group [4]
- Postoperative complications according to the Clavien-Dindo classification [5]

## **Rationale and description of the imputation of the QoR-40 scores**

Previous research has established the benefits of addressing missing data at the item level within multi-item scales to maximize information use, thereby enhancing the precision and power of statistical analyses.[6,7] However, numerous scales can result in an excessively high number of items, hindering the imputation model's capacity to generate reliable imputations. A practical resolution to this issue, as outlined by Eekhout et al.[8], involves the adoption of a parcel summary score. This method aggregates multiple items that measure similar concepts into a single score, typically by averaging available item scores, thus reducing the number of variables within the imputation model. With this method, the imputation of item scores for each scale is conducted independently.

For the imputation of QoR-40 scores, collected at four distinct time points and comprising a significant volume of data, we employed a multiple imputation approach including the parcel summary score as described above. Initially, a parcel summary score was computed for the QoR-40 at each time point. In this case the average of the available item scores per time period for each patient. Subsequent imputation was performed separately for the QoR-40 questions corresponding to each time point. The predictors included the QoR-40 questions from the time period that was imputed along with the corresponding visual analogue scale (VAS) score. From the other time points, only the parcel summary scores were used as predictors to reduce the number of variables.

The fixed predictors (included in all imputations) encompassed the following variables: randomization group, gender, body mass index, and comorbidities including cardiac arrhythmia, chronic lung disease, cerebrovascular accident, diabetes mellitus , hypertension, use of home medications, age, hospital, and ASA score. These imputed datasets were then used to calculate the overall QoR-40 scores for each time point. Notably, for the preoperative QoR-40 scores, questions 16, 17, and 18 were excluded from the final analyses due to their irrelevance in a preoperative context, leading to a high rate of non-completion by patients.

# **Supplementary results**

## **Supplementary table 1: Postoperative pain scores, expressed in numerical (pain) rating scale (NRS)**

|  | | **Epidural**  **(n=94)** | **Paravertebral**  **(n=98)** | ***P* Value^b^** | ***P* Value^c^** |
| --- | --- | --- | --- | --- | --- |
| POD1 | NRS at rest, median (IQR) | 1 (0-3) | 2 (1-3) | <.001 | <.001 |
|  | NRS during movement, median (IQR) | 2 (0-4) | 3 (2-5) | <.001 | <.001 |
|  | Localization of pain^a^, no. (%) |  |  |  |  |
|  | Thoracic | 22 (23.4) | 27 (27.6) | .367 | .440 |
|  | Abdominal | 8 (8.5) | 22 (22.4) | .004 | .018 |
|  | Drains | 16 (17.0) | 20 (20.4) | .427 | .480 |
|  | No pain | 43 (45.7) | 28 (28.6) | <.001 | <.001 |
| POD2 | NRS at rest, median (IQR) | 1 (0-2) | 2 (0-3) | .011 | .033 |
|  | NRS during movement, median (IQR) | 2 (0-4) | 3 (2-5) | .007 | .025 |
|  | Localization of pain^a^, no. (%) |  |  |  |  |
|  | Thoracic | 27 (28.7) | 23 (23.5) | .504 | .534 |
|  | Abdominal | 9 (9.6) | 20 (20.4) | .025 | .050 |
|  | Drains | 16 (27.0) | 29 (29.6) | .025 | .050 |
|  | No pain | 41 (43.6) | 25 (25.5) | .013 | .033 |
| POD3 | NRS at rest, median (IQR) | 1 (0-3) | 2 (0-3) | .245 | .315 |
|  | NRS during movement, median (IQR) | 2 (0-5) | 3 (2-4) | .158 | .239 |
|  | Localization of pain^a^, no. (%) |  |  |  |  |
|  | Thoracic | 18 (19.1) | 18 (28.4) | .760 | .760 |
|  | Abdominal | 10 (10.6) | 18 (28.4) | .159 | .239 |
|  | Drains | 18 (19.1) | 27 (27.6) | .217 | .300 |
|  | No pain | 34 (36.2) | 24 (24.5) | .037 | .067 |

Abbreviations: POD, postoperative day; IQR, interquartile range.

^a^ Based on the question ‘*Where is most pain located?*’, multiple options could be chosen.

^b^ Calculated with the χ^2^ test, in case of median (IQR) calculated with Mann-Whitney U Test

^c^ *P* Values corrected with the Benjamini Hochberg method

## **Supplementary table 2: The IPO questionnaire POD 1, 2 and 3**

|  | | **Epidural**  **(n=94)** | **Paravertebral**  **(n=98)** | ***P* Value^b^** | ***P* Value^c^** |
| --- | --- | --- | --- | --- | --- |
| POD1 | Items |  |  |  |  |
|  | 1. Worst pain since operation^a^ | 3 (1-6) | 6 (4-8) | <.001 | <.001 |
|  | 2. Least pain since operation^a^ | 0 (0-2) | 2 (0-3) | .003 | .009 |
|  | 3. Percentage of time in severe pain^b^ | 10 (0-20) | 20 (10-50) | <.001 | <.001 |
|  | 4. Pain interference with activities in bed^a^ | 3 (0-6) | 6 (3-8) | <.001 | <.001 |
|  | 5. Pain interference with breathing/coughing^a^ | 3 (1-6) | 6 (3-8) | .003 | .009 |
|  | 6. Pain interference with sleep^a^ | 0 (0-0) | 0 (0-2) | .106 | .167 |
|  | 7. Pain interference with activities out of bed^a^ | 1 (0-3) | 3 (1-5) | .006 | .015 |
|  | 8. Emotional impairment due to pain: anxious^a^ | 0 (0-0) | 0 (0-1) | .007 | .015 |
|  | 9. Emotional impairment due to pain: helpless^a^ | 0 (0-0) | 0 (0-2) | .003 | .009 |
|  | 10. Adverse effects: nausea^a^ | 0 (0-0) | 0 (0-1) | .450 | .550 |
|  | 11. Adverse effects: drowsiness^a^ | 0 (0-3) | 3 (0-6) | <.001 | <.001 |
|  | 12. Adverse effects: itching^a^ | 0 (0-0) | 0 (0-0) | .237 | .326 |
|  | 13. Adverse effects: dizziness^a^ | 0 (0-1) | 0 (0-3) | .045 | .083 |
|  | 14. Percentage of pain relief since operation^b^ | 80 (50-100) | 80 [(60-100) | .093 | .157 |
|  | 15. Participation in decision making^a^ | 8 (7-9) | 8 (5-8]) | .146 | .214 |
|  | 16. Satisfaction with pain treatment^a^ | 9 (8-10) | 8 (8-9) | .006 | .015 |
|  | Binary items, no. (%) |  |  |  |  |
|  | 17. Time spent out of bed since operation | 52 (55.3) | 52 (53.1) | .712 | .783 |
|  | 18. Wish for more pain relief | 4 (4.3) | 14 (14.3) | .011 | .022 |
|  | 19. Information on pain treatment options received | 83 (88.3) | 79 (80.6) | .448 | .550 |
|  | 20. Use of non-medicine methods for pain relief | 18 (19.1) | 19 (19.4) | .759 | .795 |
|  | 21. Chronic pain 3 or more months before operation | 14 (14.9) | 14 (14.3) | 1.000 | 1.000 |
|  | If yes: severity of chronic pain^a^ | 7 (4-8) | 5 (4-8) | .685 | .783 |
| POD2 | Items |  |  |  |  |
|  | 1. Worst pain since operation^a^ | 3 (2-7) | 6 (3-8) | <.001 | <.001 |
|  | 2. Least pain since operation^a^ | 1 (0-2) | 2 (0-3) | .002 | .006 |
|  | 3. Percentage of time in severe pain^b^ | 0 (10-30) | 20 (10-50) | <.001 | <.001 |
|  | 4. Pain interference with activities in bed^a^ | 4 (1-7) | 6 (3-8) | .003 | .007 |
|  | 5. Pain interference with breathing/coughing^a^ | 4 (1-7) | 5 (3-7) | .003 | .007 |
|  | 6. Pain interference with sleep^a^ | 0 (0-2) | 2 (0-5) | <.001 | <.001 |
|  | 7. Pain interference with activities out of bed^a^ | 2 (0-6) | 5 (2-5) | .006 | .012 |
|  | 8. Emotional impairment due to pain: anxious^a^ | 0 (0-1) | 0 (0-5) | <.001 | <.001 |
|  | 9. Emotional impairment due to pain: helpless^a^ | 0 (0-2) | 0 (0-4) | <.001 | <.001 |
|  | 10. Adverse effects: nausea^a^ | 0 (0-2] | 0 (0-0) | .669 | .775 |
|  | 11. Adverse effects: drowsiness^a^ | 1 (0-3) | 3 (1-5) | .012 | .019 |
|  | 12. Adverse effects: itching^a^ | 0 (0-2) | 0 (0-0) | .200 | .275 |
|  | 13. Adverse effects: dizziness^a^ | 0 (0-2) | 2 (0-3) | .009 | .015 |
|  | 14. Percentage of pain relief since operation^b^ | 80 (43-100) | 70 (43-80) | .001 | .003 |
|  | 15. Participation in decision making^a^ | 7 (2-8) | 8 (5-9) | .009 | .015 |
|  | 16. Satisfaction with pain treatment^a^ | 9 (7-9) | 8 (7-9) | <.001 | <.001 |
|  | Binary items, no. (%) |  |  |  |  |
|  | 17. Time spent out of bed since operation | 85 (90.4) | 84 (85.7) | .989 | .989 |
|  | 18. Wish for more pain relief | 8 (8.5) | 17 (17.3) | .048 | .070 |
|  | 19. Information on pain treatment options received | 82 (87.2) | 83 (84.7) | .479 | .585 |
|  | 20. Use of non-medicine methods for pain relief | 19 (20.2) | 25 (25.5) | .255 | .330 |
|  | 21. Chronic pain 3 or more months before operation | 14 (14.9) | 12 (12.2) | .826 | .909 |
|  | If yes: severity of chronic pain^a^ | 7 (5-8) | 7 (6-8) | .940 | .985 |
| POD3 | Items |  |  |  |  |
|  | 1. Worst pain since operation^a^ | 5 (2-7) | 6 (4-8) | .008 | .040 |
|  | 2. Least pain since operation^a^ | 1 (0-3) | 2 (1-3) | .099 | .182 |
|  | 3. Percentage of time in severe pain^b^ | 10 (0-30) | 20 (10-50) | .002 | .033 |
|  | 4. Pain interference with activities in bed^a^ | 3 (1-6) | 5 (3-7) | .005 | .037 |
|  | 5. Pain interference with breathing/coughing^a^ | 4 (2-7) | 6 (3-8) | .003 | .033 |
|  | 6. Pain interference with sleep^a^ | 1 (0-3) | 1 (0-4) | .087 | .180 |
|  | 7. Pain interference with activities out of bed^a^ | 3 (0-5) | 1 (0-4) | .654 | .899 |
|  | 8. Emotional impairment due to pain: anxious^a^ | 0 (0-1) | 0 (0-3) | .018 | .066 |
|  | 9. Emotional impairment due to pain: helpless^a^ | 0 (0-3) | 1 (0-3) | .070 | .171 |
|  | 10. Adverse effects: nausea^a^ | 0 (0-0) | 0 (0-2) | .258 | .437 |
|  | 11. Adverse effects: drowsiness^a^ | 0 (0-3) | 2 (0-4) | .025 | .079 |
|  | 12. Adverse effects: itching^a^ | 0 (0-1) | 0 (0-1) | .807 | .981 |
|  | 13. Adverse effects: dizziness^a^ | 1 (0-2) | 1 (0-3) | .396 | .622 |
|  | 14. Percentage of pain relief since operation^b^ | 90 (53-98) | 80 (60-95) | .009 | .040 |
|  | 15. Participation in decision making^a^ | 6 (2-8) | 8 (5-9) | .817 | .981 |
|  | 16. Satisfaction with pain treatment^a^ | 9 (6-10) | 8 (8-10) | .067 | .171 |
|  | Binary items, no. (%) |  |  |  |  |
|  | 17. Time spent out of bed since operation | 87 (92.6) | 90 (91.8) | .981 | .981 |
|  | 18. Wish for more pain relief | 14 (14.9) | 15 (15.3) | .974 | .981 |
|  | 19. Information on pain treatment options received | 82 (87.2) | 86 (87.8) | .954 | .981 |
|  | 20. Use of non-medicine methods for pain relief | 18 (19.1) | 29 (29.6) | .090 | .180 |
|  | 21. Chronic pain 3 or more months before operation | 15 (16.0) | 15 (15.3) | .848 | .981 |
|  | If yes: severity of chronic pain^a^ | 6 (5-8) | 7 (4-8) | .477 | .700 |

Abbreviations: POD, postoperative day; IPO, International Pain Outcomes questionnaire; IQR, interquartile range.

^a^ On a 0 – 10 scale, in median (IQR)

^b^ On a 0% – 100% scale, in median (IQR)

^c^ Binary items calculated with the χ^2^ test, continuous items (median (IQR)) calculated with Mann-Whitney U Test

^d^ P-values corrected with the Benjamini Hochberg method

## **Supplementary table 3: Intraoperative outcomes**

|  | **Epidural**  **(n=94)** | **Paravertebral**  **(n=98)** | ***P* Value^a^** | ***P* Value^b^** |
| --- | --- | --- | --- | --- |
| SMEQ score, median (IQR) | 40 (12-75) | 30 (20-49) | .164 | .389 |
| Duration of placement, median (IQR), min | 15 (12-19) | 5 (4-8) | <.001 | <.001 |
| Duration of anesthesia, mean (SD), min | 462 (87) | 471 (81) | .470 | .752 |
| Duration of induction, mean (SD), min | 51 (21) | 44 (36) | .097 | .389 |
| Duration of surgery, mean (SD), min | 404 (85) | 405 (84) | .922 | .922 |
| Duration of reversal, mean (SD), min | 9 (22) | 10 (28) | .834 | .890 |
| No. (%) with vasopressor/inotrope use | 93 (98.9) | 98 (100) | .303 | .606 |
| No. (%) with noradrenaline use | 88 (93.6) | 96 (98) | .132 | .389 |
| Consumption, mean (SD), mg | 2.0 (2.0) | 2.2 (2.1) | .518 | .753 |
| No. (%) with ephedrine use | 38 (40.4) | 36 (36.7) | .599 | .774 |
| Consumption, mean (SD), mg | 12.4 (9.3) | 11.6 (7.5) | .673 | .774 |
| No. (%) with phenylephrine use | 27 (28.7) | 34 (34.7) | .467 | .752 |
| Consumption, mean (SD), mg | 2.3 (3.5) | 1.2 (2.4) | .170 | .389 |
| Fluid balance, mean (SD), mL mg | 1636 (1169) | 1390 (1015) | .121 | .389 |
| Total opioid consumption, median (IQR), OME^^^ | 1371 (388-2442) | 1790 (345-2610) | .677 | .774 |
| Total ketanest consumption, median (IQR) | 31 (25-73) | 55 (25-80) | .158 | .389 |

Abbreviations: IQR, interquartile range; SD, standard deviation; OME, oral morphine equivalents.

^a^ Calculated with the χ^2^ test, in case of median [IQR] calculated with Mann-Whitney U Test, in case of mean (SD) calculated with Independent Samples T-Test

^b^ P-values corrected with the Benjamini Hochberg method

^^^ References used for calculating OME [9-13]

## **Supplementary table 4: Postoperative opioid consumption**

|  |  | **Epidural**  **(n=94)** | **Paravertebral (n=98)** | ***P* Value^a^** | ***P* Value^b^** |
| --- | --- | --- | --- | --- | --- |
| POD1 | Total opioid consumption, median (IQR), OME^^^ | 381 (286-477) | 58 (26-87) | <.001 | <.001 |
| POD2 | Total opioid consumption, median (IQR), OME^^^ | 382 (62-509) | 56 (24-110) | <.001 | <.001 |
| POD3 | Total opioid consumption, median (IQR), OME^^^ | 274 (30-382) | 41 (10-114) | <.001 | <.001 |

Abbreviations: POD, postoperative day; IQR, interquartile range; OME, oral morphine equivalents.

^a^ Calculated with Mann-Whitney U Test

^b^ P-values corrected with the Benjamini Hochberg method

^^^ References used for calculating OME [9-13]

## **Supplementary table 5: Mobilization**

|  | | **Epidural**  **(n=94)** | **Paravertebral**  **(n=98)** | ***P* Value^a^** |
| --- | --- | --- | --- | --- |
| POD1 | Time in chair, median (IQR) | 30 (0-60) | 25 (1-45) | .396 |
|  | No. (%) of missing values | 5 (5.3) | 11 (11.2) |  |
|  | Walking, median (IQR), number of times | 1 (0-1) | 1 (0-1) | .317 |
|  | No. (%) of missing values | 3 (3.2) | 6 (6.3) |  |
| POD2 | Time in chair, median (IQR) | 33 (20-101) | 45 (20-90) | .825 |
|  | No. (%) of missing values | 18 (19.1) | 19 (19.4) |  |
|  | Walking, median (IQR), number of times | 2 (1-2) | 2 (1-2) | .932 |
|  | No. (%) of missing values | 11 (11.7) | 13 (13.3) |  |
| POD3 | Time in chair, median (IQR) | 45 (20-93) | 58 (28-110) | .738 |
|  | No. (%) of missing values | 32 (34.0) | 32 (32.7) |  |
|  | Walking, median (IQR), number of times | 2 (1-3) | 2 (1-3) | .923 |
|  | No. (%) of missing values | 19 (20.2) | 22 (22.4) |  |

Abbreviations: POD, postoperative day; IQR, interquartile range.

^a^ Calculated with Mann-Whitney U Test

## **Supplementary table 6: Fluid balance and vasopressor consumption intra- and postoperative**

|  |  | **Epidural**  **(n=94)** | **Paravertebral (n=98)** | **P Value^a^** | ***P* Value^b^** |
| --- | --- | --- | --- | --- | --- |
| POD 0 | Vasopressor use | 35 (37.2) | 24 (24.4) | .084 | 0.504 |
|  | Noradrenaline use |  |  |  |  |
|  | Consumption, mean (SD), mg | 1.3 (1.5) | 1.4 (2.8) |  |  |
|  | Fluid balance, mean (SD), mL | 365 (707) | 323 (810) | .702 | 1.000 |
| POD 1 | Vasopressor use | 36 (38.3) | 13 (13.3) | <.001 | <.001 |
|  | Noradrenaline use |  |  |  |  |
|  | Consumption, mean (SD), mg | 2.7 (2.7) | 2.1 (2.3) | .538 | .926 |
|  | Fluid balance, mean (SD), mL | 778 (1305) | 765 (1069) | .939 | 1.000 |
| POD 2 | Vasopressor use | 8 (8.5) | 4 (4.1) | .243 | .852 |
|  | Noradrenaline use |  |  |  |  |
|  | Consumption, mean (SD), mg | 5.4 (3.8) | 2.7 (3.9) | .284 | .852 |
|  | Fluid balance, mean (SD), mL | 522 (985) | 416 (999) | .463 | .926 |
| POD 3 | Vasopressor use | 4 (4.3) | 4 (4.1) | 1.000 | 1.000 |
|  | Noradrenaline use |  |  |  |  |
|  | Consumption, mean (SD), mg | 4.6 (5.4) | 5.5 (9.2) | .891 | 1.000 |
|  | Fluid balance, mean (SD), mL | 286 (1034) | 259 (1108) | .864 | 1.000 |
| Cumulative fluid balance | Fluid balance, mean (SD), mL | 1970 (2473) | 1750 (2387) | .540 | .926 |

Abbreviations: POD, postoperative day; SD, standard deviation.

^a^ Calculated with the χ^2^ test, in case of mean (SD) calculated with Independent Samples T-Test

^b^ P-values corrected with the Benjamini Hochberg method

# **References**

[1] Wensing AGCL, van Cuilenborg VR, Breel JS, Heineman DJ, Hermanides J, Hollmann MW, Ten Hoope W. Psychometric evaluation of the Dutch 40-item Quality-of-Recovery scale. Br J Anaesth. 2022 Jan;128(1):e6-e8. doi: 10.1016/j.bja.2021.09.022.

[2] Rothaug J, Zaslansky R, Schwenkglenks M, Komann M, Allvin R, Backström R, Brill S, Buchholz I, Engel C, Fletcher D, Fodor L, Funk P, Gerbershagen HJ, Gordon DB, Konrad C, Kopf A, Leykin Y, Pogatzki-Zahn E, Puig M, Rawal N, Taylor RS, Ullrich K, Volk T, Yahiaoui-Doktor M, Meissner W. Patients' perception of postoperative pain management: validation of the International Pain Outcomes (IPO) questionnaire. J Pain. 2013 Nov;14(11):1361-70. doi: 10.1016/j.jpain.2013.05.016.

[3] Sauro J, Dumas JC. Comparison of three one-question, post-task usability questionnaires. *Proceedings of the SIGCHI Conference on Human Factors in Computing Systems*, New York, NY, USA: ACM, Apr. 2009, pp. 1599–1608. doi: 10.1145/1518701.1518946.

[4] Blencowe NS, Strong S, McNair AG, Brookes ST, Crosby T, Griffin SM, Blazeby JM. Reporting of short-term clinical outcomes after esophagectomy: a systematic review. Ann Surg. 2012 Apr;255(4):658-66. doi: 10.1097/SLA.0b013e3182480a6a. PMID: 22395090.

[5] Dindo D, Demartines N, Clavien PA. Classification of surgical complications: a new proposal with evaluation in a cohort of 6336 patients and results of a survey. Ann Surg. 2004 Aug;240(2):205-13. doi: 10.1097/01.sla.0000133083.54934.ae. PMID: 15273542; PMCID: PMC1360123.

[6] Eekhout I, de Vet HCW, Twisk JWR, et al. Missing data in a multi-item instrument were best handled by multiple imputation at the item score level. J Clin Epidemiol. 2014; 67: 335–342. doi: 10.1016/j.jclinepi.2013.09.009.

[7] Gottschall AC, West SG, Enders CK. A comparison of item-level and scale-level multiple imputation for questionnaire batteries. Multivariate Behav Res. 2012; 47: 1–25. doi: 10.1080/00273171.2012.640589.

[8] Eekhout I, de Vet HCW, de Boer MR, Twisk JWR, Heymans MW. Passive imputation and parcel summaries are both valid to handle missing items in studies with many multi-item scales. Stat Methods Med Res. 2018; 27(4): 1128–1140. doi: 10.1177/0962280216654511.

[9] Nielsen S, Degenhardt L, Hoban B, Gisev N. A synthesis of oral morphine equivalents (OME) for opioid utilisation studies. Pharmacoepidemiol Drug Saf. 2016;25(6):733-7.

[10] Toombs JD, Kral LA. Methadone treatment for pain states. Am Fam Physician. 2005;71(7):1353-8.

[11] Prevention CfDCa. CALCULATING TOTAL DAILY DOSE OF OPIOIDS FOR SAFER DOSAGE. In: https://www.cdc.gov/drugoverdose/pdf/calculating_total_daily_dose-a.pdf, editor.

[12] Walker PW, Palla S, Pei BL, Kaur G, Zhang K, Hanohano J, et al. Switching from methadone to a different opioid: what is the equianalgesic dose ratio? J Palliat Med. 2008;11(8):1103-8.
[13] Pain Management Education at UCSF https://pain.ucsf.edu/opioid-analgesics/calculation-oral-morphine-equivalents-ome
